# Trends in use of antiseizure medication and treatment pattern during the first trimester in the German Embryotox cohort

**DOI:** 10.1038/s41598-024-83060-9

**Published:** 2024-12-23

**Authors:** Maria Hoeltzenbein, Sofia Slimi, Anne-Katrin Fietz, Katarina Dathe, Christof Schaefer

**Affiliations:** Embryotox Center of Clinical Teratology and Drug Safety in Pregnancy, Institute of Clinical Pharmacology and Toxicology, Charité – Universitätsmedizin Berlin, corporate member of Freie Universität Berlin, Humboldt-Universität zu Berlin, Berlin, Germany

**Keywords:** Antiseizure medication, epilepsy, teratogen, pregnancy, valproate, Neurology, Psychiatric disorders, Reproductive disorders

## Abstract

Because of their developmental toxicity, some antiseizure medication (ASM) should be avoided during pregnancy. This may lead to discontinuation or switching of ASM after recognition of pregnancy, but some of these changes may be suboptimal. Trends in ASM use at conception were analyzed in 3,763 pregnancies prospectively ascertained by a teratology information service in Germany between 2000 and 2018. Focusing on women with epilepsy (*n* = 2,395), changes of treatment pattern during the 1st trimester were evaluated. There was an increase in women using ASMs for non-epilepsy indications from 19% in 2000 to 39% in 2018. Although a shift from non-recommended teratogenic ASMs to recommended ASMs was observed, 13% of women were still receiving non-recommended ASMs in early pregnancy at the end of the study period. Among women with epilepsy, ASM regimen remained unchanged during the 1st trimester in 90%, 6% switched to other ASMs, and only 4% discontinued. Valproate, oxcarbazepine, and topiramate were more likely to be discontinued or switched than other ASMs. This first analysis of treatment pattern in ASM exposed pregnancies in Germany confirms the general trend towards less teratogenic and newer ASMs. However, it remains questionable whether women still using non-recommended ASMs are refractory to lower-risk ASMs or disregard treatment guidelines and risk minimization measures.

**Supplementary Information:**

The online version contains supplementary material available at 10.1038/s41598-024-83060-9.

## Introduction

Pregnancies increasingly occur in women with chronic conditions, which may require a change in pre-pregnancy medication to alternatives with lower risk for the unborn child. This has been widely discussed for hypertension with replacement of fetotoxic ACE-inhibitors or ARBs^1^ or rheumatic diseases with replacement of MTX or mycophenolate^2^. The same applies to valproate, a first-line treatment for generalized seizures except in women of childbearing age. Repeated warnings have been released against the use of valproate in women of childbearing age since 2014 ^3^. At the same time, newer ASMs with fewer side effects and better tolerability have become available. However, experience in pregnancy is often very limited with the exception of lamotrigine and levetiracetam, both regarded as drugs of choice during pregnancy^4,5^.

Our study aims to assess ASM exposure pattern in prospectively ascertained pregnancies.

## Methods

The Embryotox Center of Clinical Teratology and Drug Safety in Pregnancy (Embryotox) at the Charité-Universitätsmedizin Berlin, Germany offers advice on drug therapy during pregnancy. Approximately 14,000 requests from health care professionals and patients are answered every year. At the first consultation, drug exposures, treatment indications, maternal medical history and obstetric and family history are recorded after informed consent. About 8 weeks after the expected date of delivery, the pregnancy is followed up via mailed questionnaires and structured telephone interviews. Annually, about 4,000 drug exposed pregnancies and their course and outcome are recorded and critically evaluated. Further information on the methodology, which was adapted to the recommendations of the Strengthening of the Reporting of Observational studies in Epidemiology (STROBE) statement has been published elsewhere^6^.

### Study design

The study is an observational study based on prospectively ascertained pregnancies identified by risk consultation at Embryotox between 1.1.2000 and 31.12.2018. Prospective in this context means that neither pregnancy outcome nor pathological results from prenatal diagnostics were known at the time of enrolment.

The study cohort included pregnancies with at least one ASM at conception. Gestational age was calculated either based on ultrasound measures during the first trimester or, if not available, using the first day of the last menstruation. First trimester exposure was defined as exposure between gestational week 2 + 0 days (presumed date of conception) and gestational week 12 + 6 days after the first day of last menstrual period (LMP).

For definition of ASM see Table S1. Classification of treatment indication was based on MeDRA-Codes. Pregnancies with use of different ASMs for both epilepsy and other indications, were assigned to the epilepsy cohort.

Monotherapy was defined as the use of one ASM at a given time period, e.g. at conception for the ASM and epilepsy cohorts and at conception, gestational week 8 and 12 for analysis of treatment changes during pregnancy. The use of more than one ASM (regardless of treatment indication) was considered as polytherapy. Treatment changes were specified as discontinuation, change or addition of one or more different ASMs.

**Fig. 1:**
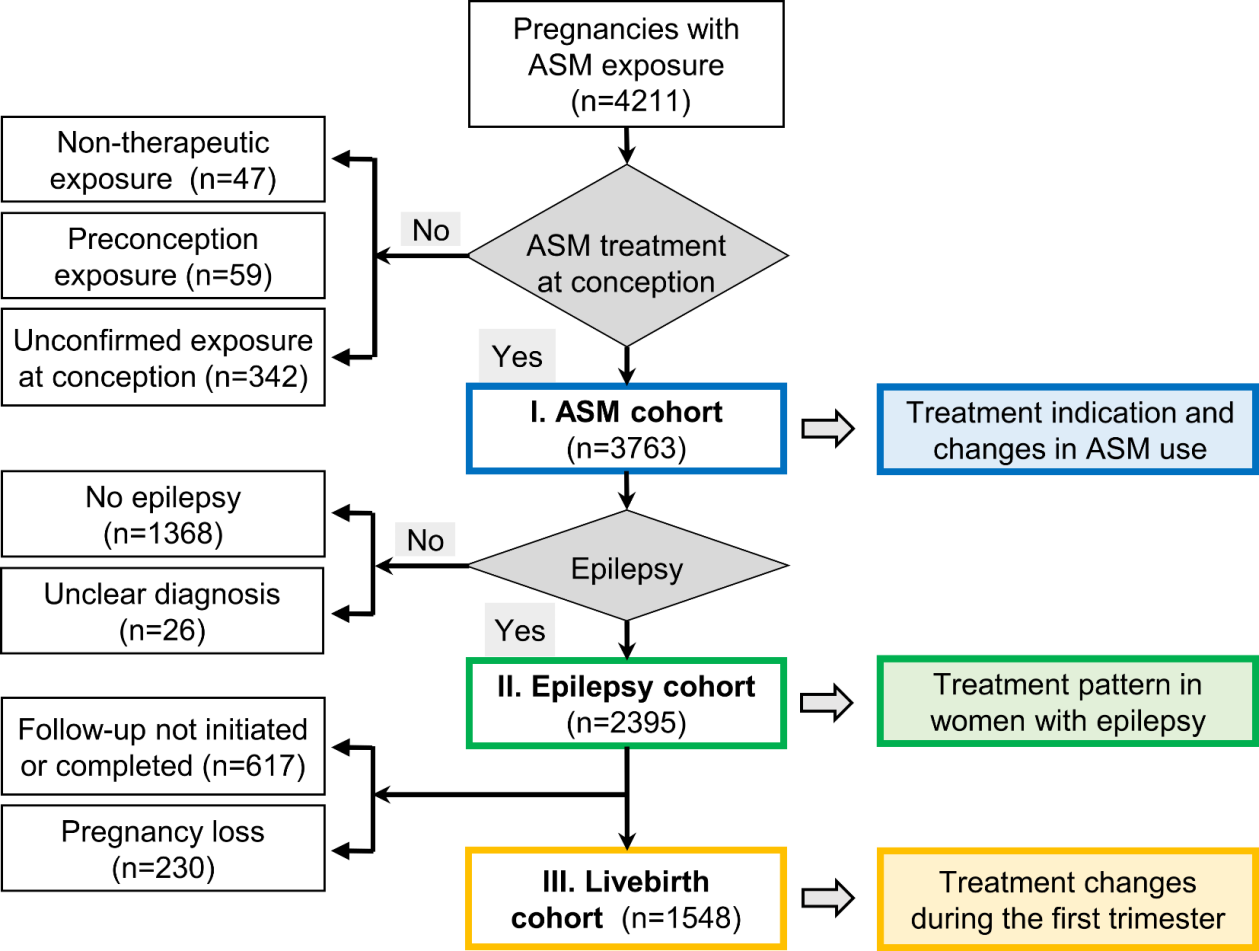
Overview on study design and selection of study cohorts from prospectively ascertained pregnancies with maternal antiseizure medication (ASM) at conception from 2000–2018.

The aim of the study was to analyze trends of ASM use including treatment indications in pregnant women during the study period 2000–2018. For time trends of ASM use of different teratogenic risks and the proportion of mono- and polytherapy, all women with epilepsy on ASM at conception were analyzed (epilepsy group). Changes in treatment during gestational course were assessed in the subgroup of women with epilepsy and livebirth (Fig. 1).

### Statistical analysis

Data exported from the Embryotox database were descriptively analyzed. Trends over time for use of ASM were analyzed using Chi-squared test. For the analysis of paired data, e.g. comparison of ASM proportion during pregnancy, McNemar-test was used. All analyses were performed using R Statistical Software (version 3.6.1, R Core Team, Vienna, Austria^7^). Significance level was set at 0.05. SankeyMATIC (Bogart S., www.sankeymatic.com) was used for the Sankey diagram.

### Study protocol / Ethics

The study protocol was approved by the Ethics Committee of the Charité-Universitätsmedizin Berlin (EA4/015/20, approved on 7.2.2020). The study was registered in the German Clinical Trial register (DRKS00021001) and listed in the WHO International Clinical Trials Registry Platform. The study was performed in accordance with the relevant guidelines and regulations. Informed consent was obtained from all participants included in the study.

## Results

This observational study evaluates prospectively recorded pregnancies exposed to ASM at conception.

### ASM exposure and treatment indication at conception (ASM cohort)

**Fig. 2:**
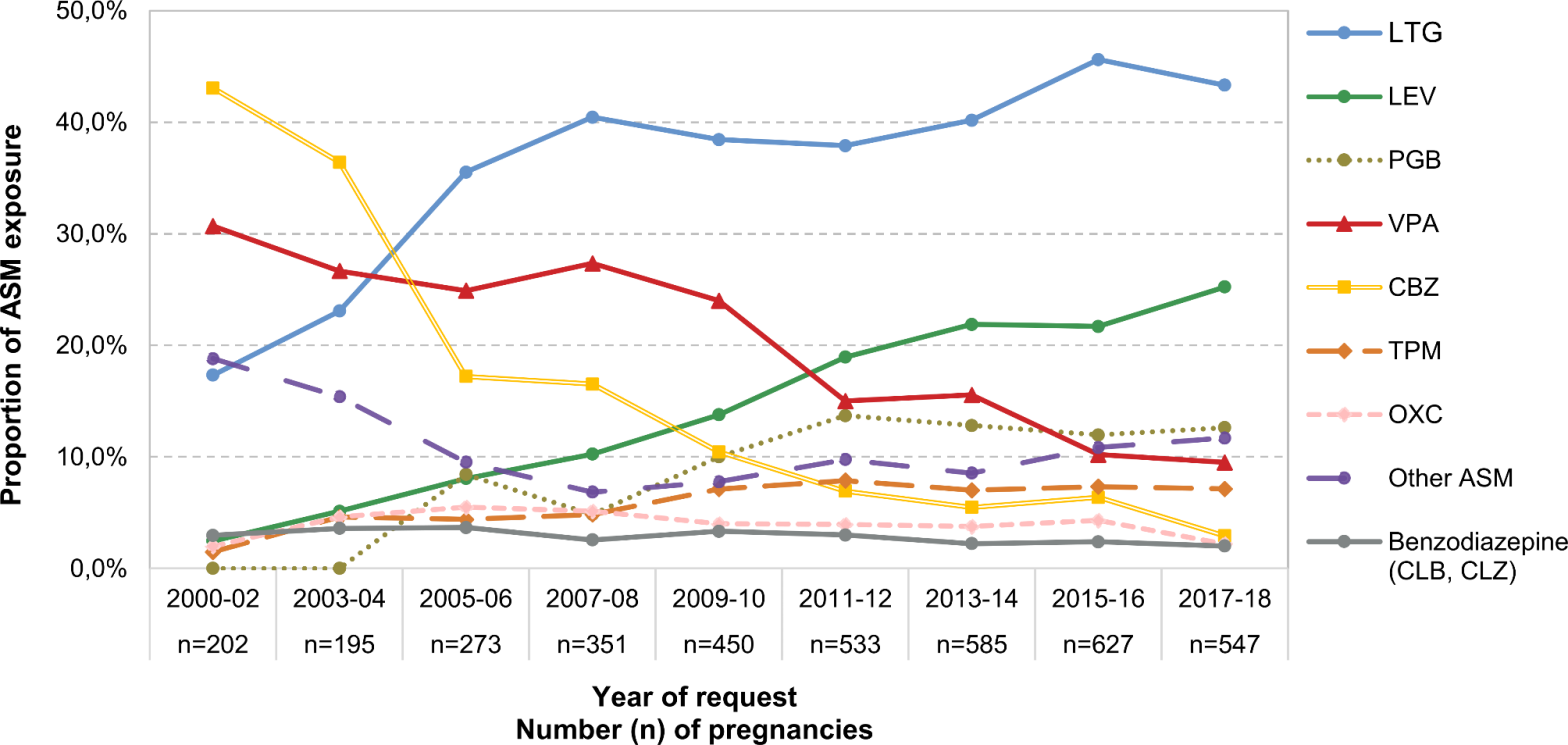
Proportion of ASM exposure at conception from 2000–2018 (ASM cohort, *n* = 3763) in 2-year intervals. For polytherapy, each ASM was regarded separately, thus proportions do not add up to 100%.

During the study period, a total of 3,763 women received an ASM at the beginning of pregnancy (Fig. 1), corresponding to 3.4% of all pregnant women reported to Embryotox during this period. Time trends of individual ASM use independent of treatment indication are shown in Fig. 2.

**Fig. 3:**
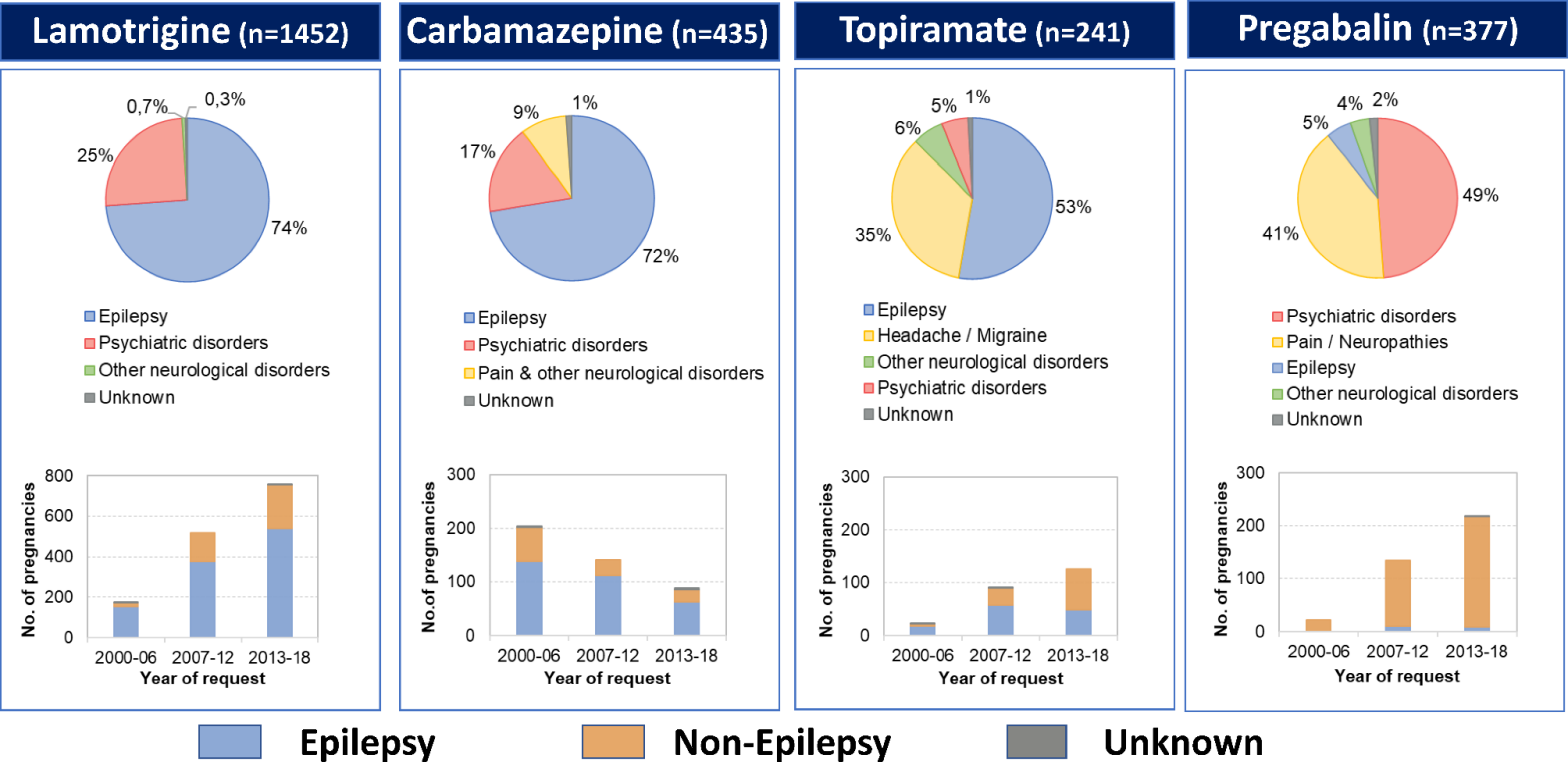
Changes in use and treatment indication for selected ASM also used for non-epilepsy indications from 2000—2018.

Over time, there was a significant increase in use of ASM for non-epilepsy indications from 23.3% in 2000–2002 to 38.8% in 2017–2018 (*p* < 0.001). The five most common ASM used by women without epilepsy were lamotrigine, pregabalin, valproate, carbamazepine and topiramate. 34.6% of women on valproate were treated for non-epilepsy conditions (mostly bipolar or psychotic disorders) and the proportion of these increased significantly over time (from 14,5% to 38,5%, *p* = 0.003). For lamotrigine the proportion of women with non-epilepsy indication increased from 10.2 to 28.4% (*p* < 0.001). Further details of time trends for the most frequently used ASM for non-epilepsy indications are shown in Fig. 3.

### ASM exposure in women with epilepsy (Epilepsy cohort)

**Fig. 4:**
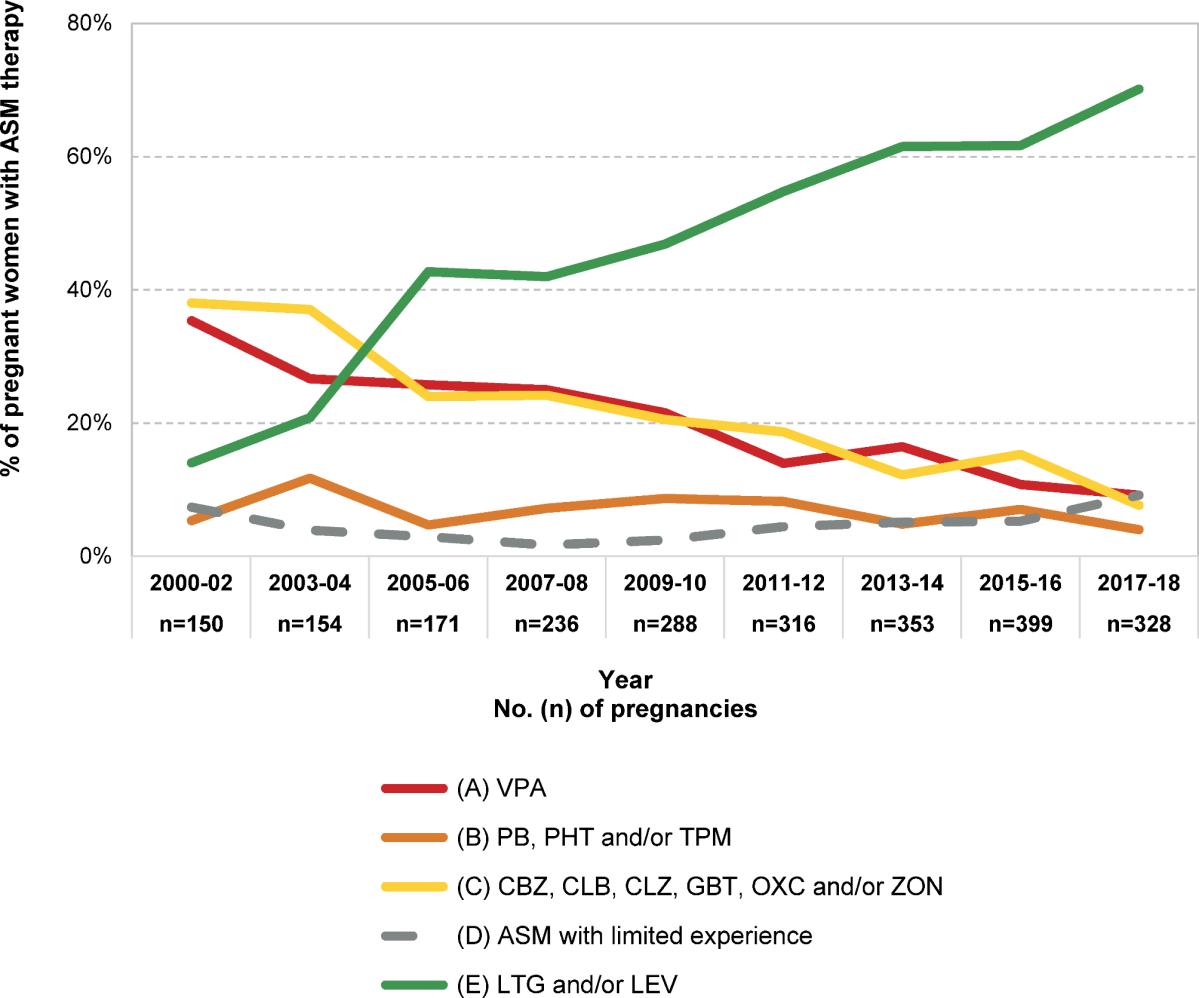
ASM use at conception by risk group in women with epilepsy. Hierarchical classification according to ASM with the highest teratogenic risk in pregnancy. Pregnancies with use of an ASM that could not be assigned to groups A to C were summarized as ASM with limited experience (group D). Women using only LTG and/or LEV were included in group E.

More than half of the ASM-exposed women were treated for epilepsy (*n* = 2,395/3,763; 63.6%). Changes over time in ASM use at conception in women with epilepsy are shown in Figure S1 and S2. Exposed pregnancies were assigned to one of four different ASM risk groups (Fig. 4). In cases of polytherapy, classification was based on the ASM with the highest teratogenic potential^8^. There was a significant increase in women treated exclusively with lamotrigine and/or levetiracetam from 14% in 2000–2002 to 70.1% in 2017–2018 (*p* < 0.001). The proportion of pregnancies exposed to valproate decreased significantly (35.3% vs. 9.1% *p* < 0.001), whereas the proportion of ASMs in group B remained almost unchanged (5.3% vs. 4%, *p* = 0.66). At the end of the study period a proportion of 13.1% of women received treatment with a high-risk ASM (group A and B) and 9.1% were treated with an ASM with limited experience (group D).

### Mono- and polytherapy

76.4% of women on ASM at conception (1,830/2,395) were receiving monotherapy. The most commonly used monotherapies were lamotrigine (*n* = 751), levetiracetam (*n* = 338), valproate (*n* = 293) and carbamazepine (*n* = 218), accounting for 87.4% of all monotherapies. An overview of monotherapies and different combination therapies is given in Figure S3.

Polytherapy with ASM was required in 565/2,395 pregnancies (23.6%), with two ASM needed in 20.2% and three or more ASM in 3.4%. There was a non-significant trend towards an increase in polytherapy during the study period. Time trends for individual ASM are shown in Figure S4.

### Treatment changes during the first trimester

Among women with epilepsy and complete information on course of treatment, ASM regimen remained unchanged during the 1st trimester in 90%, 6% switched to other ASMs, and 4% discontinued an ASM. Only 10 women (0.6%) received an additional ASM until gestational week 12. Changes in ASM therapy were more frequent in women on polytherapy than in those on monotherapy (Table 1). Valproate was the drug most frequently involved in treatment changes (Table 2). For women on monotherapy at conception treatment changes during the first trimester are shown in Figure S5.

**Table 1:** Treatment changes during the first trimester stratified by mono- and polytherapy in women with epilepsy and livebirth and complete treatment information (*n* = 1,506).

Treatment at gestational week 2	Treatment changes until gestational week 12	Proportion (*n*=)
Monotherapy (*n* = 1177)	**Continued** Discontinuation of therapy* Treatment change** * Switching* * AED added*	**93**,**3% (1098)** ** 4**,**4% (52)** ** 2**,**3% (27)** * 1*,*8% (20)* * 0*,*6% (7)*
Polytherapy (*n* = 329)	**Continued** Discontinuation of therapy* Treatment change** * Reduction in number of ASM to* * Within polytherapy* * To monotherapy*	**79**,**9% (263)** ** 2**,**1% (7)** ** 17**,**9% (59)** * 3% (10)* * 12*,*5% (41)*

*Discontinuation of all ASM.

**Treatment change: switch to other ASM, reduction of number of ASM or ASM added in polytherapy.

**Table 2:** Treatment changes during the first trimester in women with epilepsy and livebirth by frequently used ASM (*n* = 1,548).

Antiseizure medication	LTG	LEV	VPA	CBZ	TPM	OXC
**Treatment at conception (GW 2), n**	716	406	259	197	86	76
**Treatment at GW 8 (relative to GW 2)**						
Continued	98.0%	96.6%	83.0%	89.3%	88.4%	89.5%
Discontinued	0.6%	2.7%	10.4%	5.1%	4.7%	7.9%
Replaced	0.1%	0%	4.2%	2.0%	3.5%	1.3%
Unkown	1.3%	0.7%	2.3%	3.6%	3.5%	1.3%
**Treatment at GW 12 (relative to GW 2)**						
Continued	97.1%	95.3%	74.9%	82.7%	80.2%	78.9%
Discontinued	1.4%	3.7%	13.9%	8.6%	11.6%	15.8%
Replaced	0.1%	0%	6.2%	2.5%	3.5%	2.6%
Unkown	1.4%	1.0%	5.0%	6.1%	4.7%	2.6%

GW, gestational week; LTG, lamotrigine. LEV, levetiracetam. VPA, valproate, CBZ, carbamazepine; TPM, topiramate; OXC, oxcarbazepine.

## Discussion

This first analysis of ASM use in pregnant women in Germany confirms observations from other countries of increasing use of lamotrigine and levetiracetam and decreasing rates of ASM with higher teratogenic potential^9,10^. Compared with population-based data from the Netherlands where 0.36% of all pregnancies are exposed to ASM^10^, 3.4% of our consultations on drugs in pregnancy concern ASM use, thus confirming the unchanged need for counselling.

Consistent with data from UK^11^, the use of ASM for non-epilepsy indications increased, particularly for lamotrigine, topiramate and pregabalin. The significant rise in non-epilepsy indications for lamotrigine from 10.2 to 28.4% may be explained by its preference over teratogenic lithium for the treatment of bipolar disorder^12^. Increased use of topiramate was noted mainly in women with migraine, whereas use for epilepsy decreased after the first signals of teratogenicity^13,14^. The increased usage of pregabalin for non-epilepsy disorders in our study was rather striking (Fig. 3) considering the ongoing discussion about safety in pregnancy^15^. Indications were mostly psychiatric disorders and neuropathic pain which is in line with the general trend observed with pregabalin in Germany^16^.

The decrease in non-recommended teratogenic ASM in our study is mainly driven by a reduction among women with epilepsy. The second most striking decrease after VPA was observed for carbamazepine reflecting its general decline due to adverse effects and high interaction potential^16^. In addition, a similar decline was seen for other ASM no longer considered as first line drugs for epilepsy (Figure S2).

In contrast to the declining use of valproate in women with epilepsy, we observed an increase for non-epilepsy disorders confirming the need to promote awareness of the drug’s risk among psychiatrists and other health care professionals ^17 3^. Considering the overall high rates of unplanned pregnancies of up to 65% in women with epilepsy^18^, it is important to regularly address family planning and reproductive issues like contraception.

Among women with epilepsy there was a significant trend towards use of the recommended ASMs lamotrigine and levetiracetam. Since 2011/12 more than half of women with epilepsy have been treated exclusively with recommended ASMs. In addition, an increasing use of newer ASMs with limited or no experience in pregnancy was seen^19^.

More than 2/3rd of women on ASM in our epilepsy cohort were on monotherapy when getting pregnant, mainly lamotrigine, valproate and carbamazepine. Overall there was a non-significant trend towards polytherapy during the study period (*p* = 0.06). The rate of polytherapy (23.6%) was higher than the 10.3% or 12% described in recent studies from the Netherlands^10^ and UK^11^, which might be related to the inclusion of non-epilepsy indications in their studies. The proportion of polytherapy significantly increased for oxcarbazepine and decreased for levetiracetam (Figure S4).

The most frequent dual therapy was lamotrigine plus levetiracetam, both drugs recommended for use in pregnancy. Apart from levetiracetam, topiramate and oxcarbazepine were most commonly used in polytherapy. As most studies focus on monotherapy^5^, pregnancy outcome data are scarce for almost all ASM combinations, even for the common combination of lamotrigine plus levetiracetam^20^.

Changes or even discontinuation of ASM treatment during pregnancy or after recognition of pregnancy is often caused by fear of teratogenicity. As expected, the highest discontinuation rate was observed for valproate (Table 2). For those women who continued with VPA, we do not know if less teratogenic treatment options were not available or if the pregnancy prevention program was disregarded. Future studies should analyze the reasons for valproate continuation and possible strategies to reduce exposure during pregnancy.

Most women with epilepsy in our cohort continued their treatment - as commonly recommended^21^. A recent study showed that pregnancy was a common reason for self-discontinuation of ASM therapy^22^. However, changing treatment in early pregnancy may expose the patient to the risk of seizures and overlapping (transient dual) therapy may increase the risk of teratogenicity^23^. Therefore, selection of an appropriate treatment regimen is mandatory for women planning to become pregnant and, in view of unplanned pregnancies, for all women of childbearing age. The ACOG recommends that reproductive issues should be regularly addressed in patients with seizure disorders beginning in adolescence^24^. To our knowledge, similar recommendations are still missing for non-epilepsy indications.

### Strength and Limitations

A major advantage of our approach compared to registry studies based on insurance data is the rather detailed information on treatment changes during pregnancy. All pregnancies including those with fetal losses were analyzed. However, data on epilepsy type and details of seizure frequency were not routinely collected nor was ASM dose assessed in this study. We have reported p-values in order to enable assessment of the strength of evidence^25^. As we did not adjust for multiple testing false positive signals cannot be ruled out. Further strengths and limitations of observational studies on pregnancy outcome have been discussed in detail by Schaefer et al.^6^.

Although the regional distribution of enrolled women was representative of the female population of childbearing age in Germany^26^, the Embryotox cohort may not be representative of all pregnant women in Germany.

## Conclusions

Despite several regulatory measures taken in the last decade, there is a need to improve preconception counselling to minimize ASM treatment changes during pregnancy. Understanding of the risks associated with ASM in pregnancy needs to be improved, particularly among healthcare professionals dealing with non-seizure indications.

## Electronic Supplementary Material

Below is the link to the electronic supplementary material.

Supplementary Material 1

## Data Availability

The data that support the findings of this study are available on request from the corresponding author. The data are not publicly available due to privacy or ethical restrictions.

## Abbreviations

ASM: Antiseizure medication
ACE-inhibitor: Angiotensin converting enzyme inhibitor
ARB: Angiotensin receptor blocker
BfArM: German federal institute for drugs and medical devices
CI: Confidence interval
EURAP: European and international registry of antiepileptic drugs in pregnancy
GW: Gestational week
LEV: Levetiracetam
LMP: Last menstrual period
LTG: Lamotrigine
MedDRA: Medical dictionary for regulatory activities
OR: Odds ratio
OXC: Oxcarbazepine
PB: Phenobarbital
PGB: Pregabalin
PHT: Phenytoin
STROBE: Strengthening of the reporting of observational studies in epidemiology
TPM: Topiramate
UK: United Kingdom
VPA: Valproate
ZON: Zonisamide
