# Author Correction: Trends in use of antiseizure medication and treatment pattern during the first trimester in the German Embryotox cohort

**DOI:** 10.1038/s41598-025-89488-x

**Published:** 2025-02-18

**Authors:** Maria Hoeltzenbein, Sofia Slimi, Anne-Katrin Fietz, Katarina Dathe, Christof Schaefer

**Affiliations:** Embryotox Center of Clinical Teratology and Drug Safety in Pregnancy, Institute of Clinical Pharmacology and, Charité – Universitätsmedizin Berlin, corporate member of Freie Universität Berlin, Humboldt-Universität zu Berlin, Berlin, Germany

Correction to: *Scientific Reports* 10.1038/s41598-024-83060-9, published online 23 December 2024.

The Funding section in the original version of this Article was incomplete.

Open Access funding enabled and organized by Projekt DEAL.

Now reads:

This work was performed with financial support from the German Federal Institute for Drugs and Medical Devices (BfArM). Open Access funding enabled and organized by Projekt DEAL.

The original Article has been corrected.

