# Supplementary material for: Trends in use of antiseizure medication and treatment pattern during the first trimester in the German Embryotox cohort
Source: Sci Rep. 2024 Dec 23;14:30585. doi: 10.1038/s41598-024-83060-9 (PMC11666772; doi:10.1038/s41598-024-83060-9)
Supplement: Supplementary file 1 — Supplementary Material 1 [file 41598_2024_83060_MOESM1_ESM.docx]

**Trends in Use of Antiseizure Medication and Treatment Pattern during the first Trimester in the German Embryotox Cohort**

Maria Hoeltzenbein*^1^, Sofia Slimi^1^, Anne-Katrin Fietz^1^, Katarina Dathe^1^, Christof Schaefer^1^

^1^ Charité – Universitätsmedizin Berlin, corporate member of Freie Universität Berlin, Humboldt-Universität zu Berlin, Institute of Clinical Pharmacology and Toxicology, Embryotox Center of Clinical Teratology and Drug Safety in Pregnancy, Berlin, Germany

*Address for correspondence:

PD Dr. med. Maria Hoeltzenbein

Charité – Universitätsmedizin Berlin

Embryotox Center of Clinical Teratology and Drug Safety in Pregnancy

Augustenburger Platz 1

D - 13353 Berlin

Germany

Tel.: +49 (0) 30 450 525 702

Fax: +49 (0) 30 450 525 902

maria.hoeltzenbein@charite.de

# Supporting information

## Figure S1: ASM use at conception in women with epilepsy


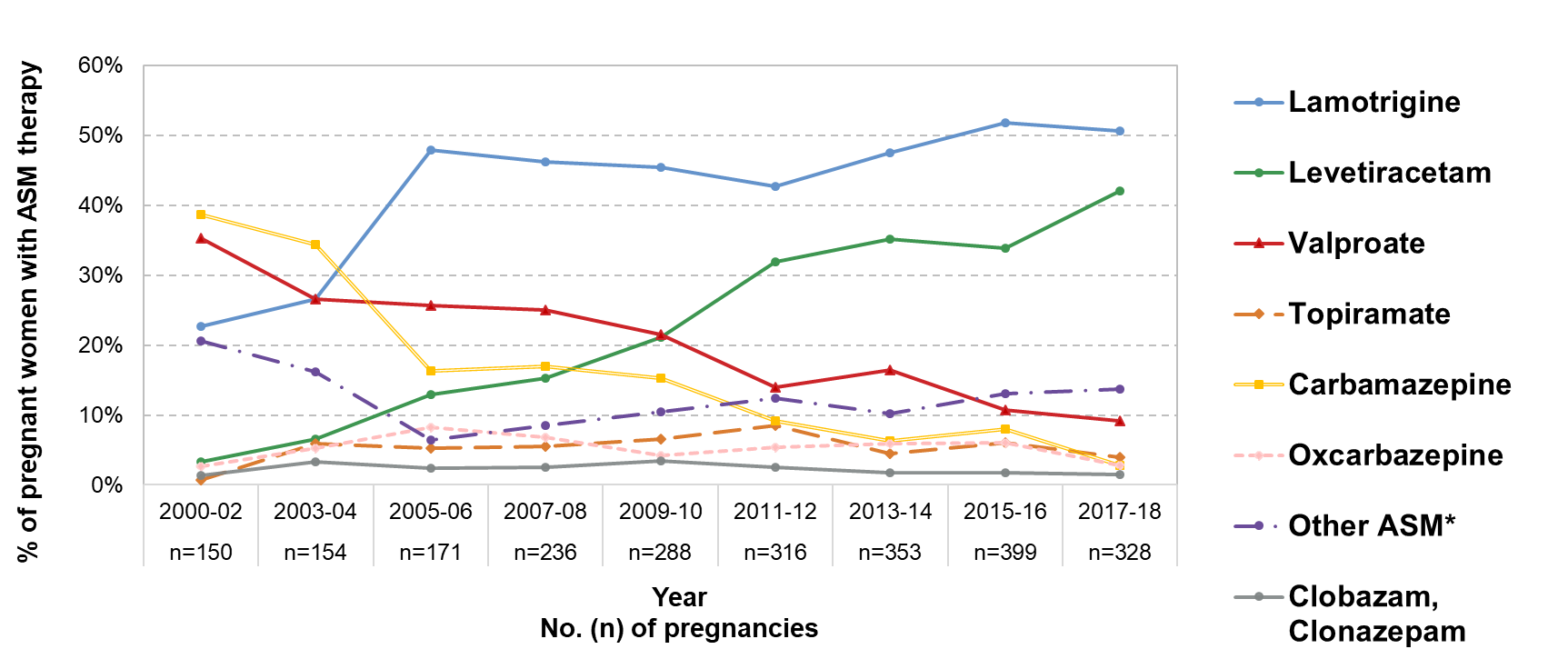


Figure S1: ASM use at conception in women with epilepsy from 2000 - 2018 (epilepsy cohort, n = 2,395). Proportion of exposures with ASM in relation to all pregnancies with epilepsy. For women with ASM polytherapy each ASM was considered separately, thus addition of percentages does not add up to 100%

*Details of other ASMs are shown in Figure S2

ASM, antiseizure medication, LTG, Lamotrigine; LEV, levetiracetam, VPA, valproate; TPM, topiramate; CBZ, carbamazepine; OXC, oxcarbazepine; CLB, Clobazam; CLZ, clonazepam.

## Figure S2: Use of other ASM for treatment of epilepsy


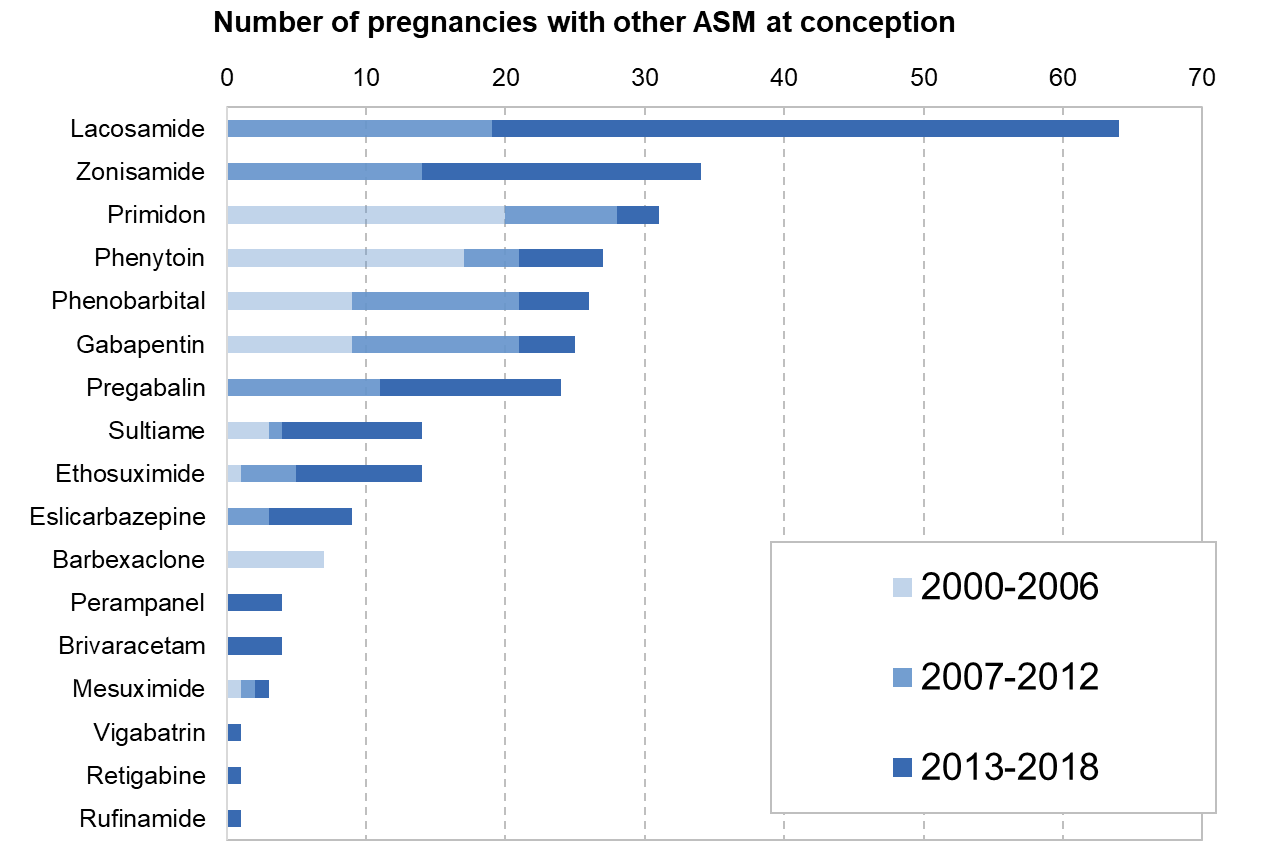


Figure S2: Less commonly used ASMs (”other ASMs” in Figure 2) in pregnancies of women with epilepsy.

## Figure S3: Mono and polytherapy in women with epilepsy at conception


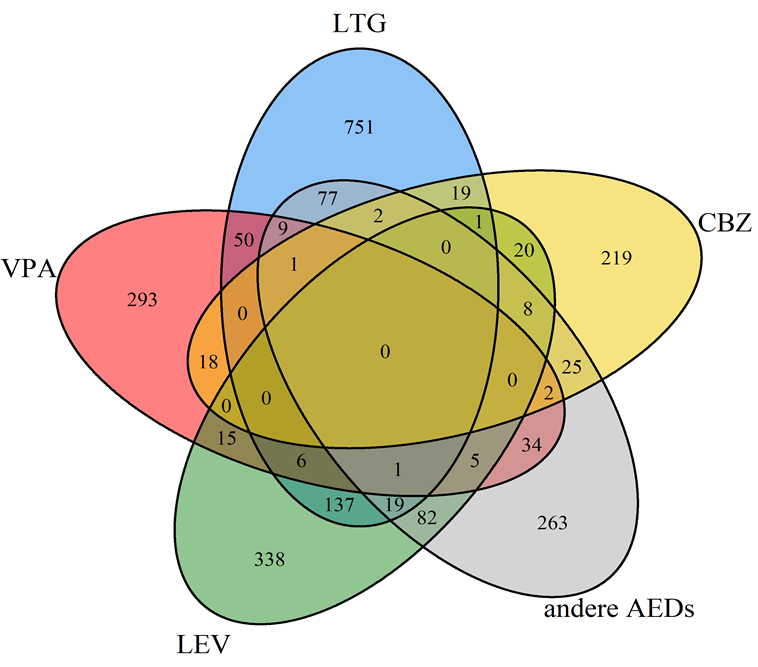


Figure S3: Mono- and polytherapy in women with epilepsy (n=2,395)

Number of pregnancies with monotherapy and polytherapy with carbamazepine (CBZ), lamotrigine (LTG), levetiracetam (LEV) and valproate (VPA). In the group of women with other ASM grey) 62 were using more than one other ASM.

## Figure S4: Proportion of polytherapy in pregnant women with epilepsy over time


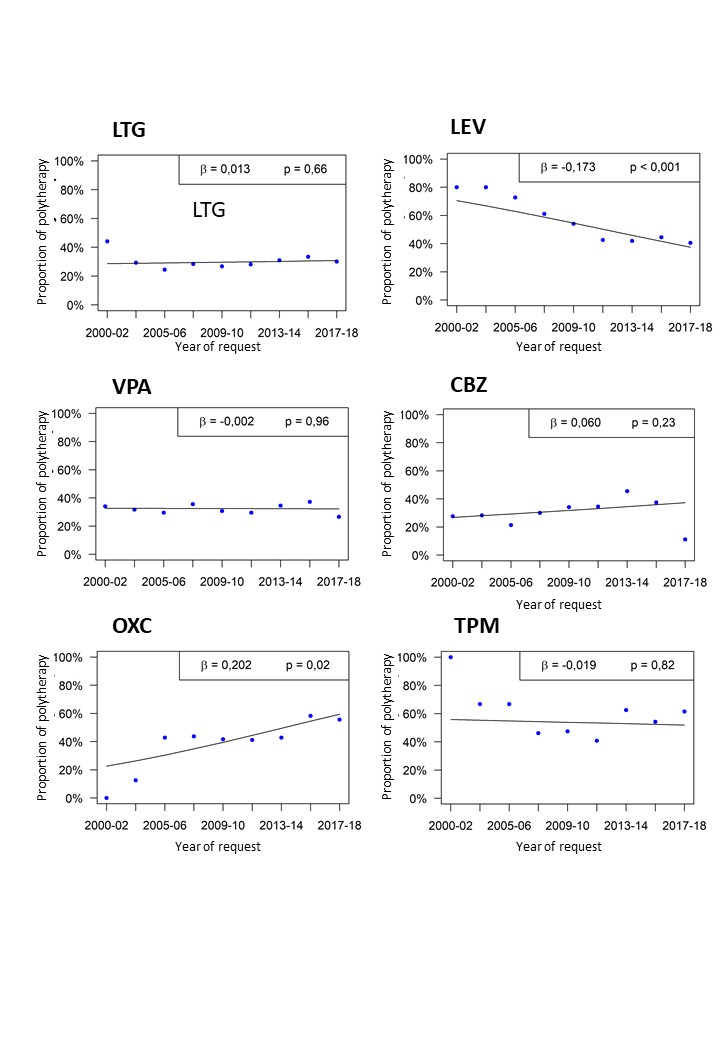


Figure S4: Time trends in polytherapy rates in women with epilepsy at conception for selected ASM (n=2,395).

LTG, lamotrigine; LEV, levetiracetam; VPA, valproate; CBZ, carbamazepine, OXC, oxcarbazepine, TPM, topiramate

## Figure S5: Treatment changes during the first trimester in women with initial monotherapy


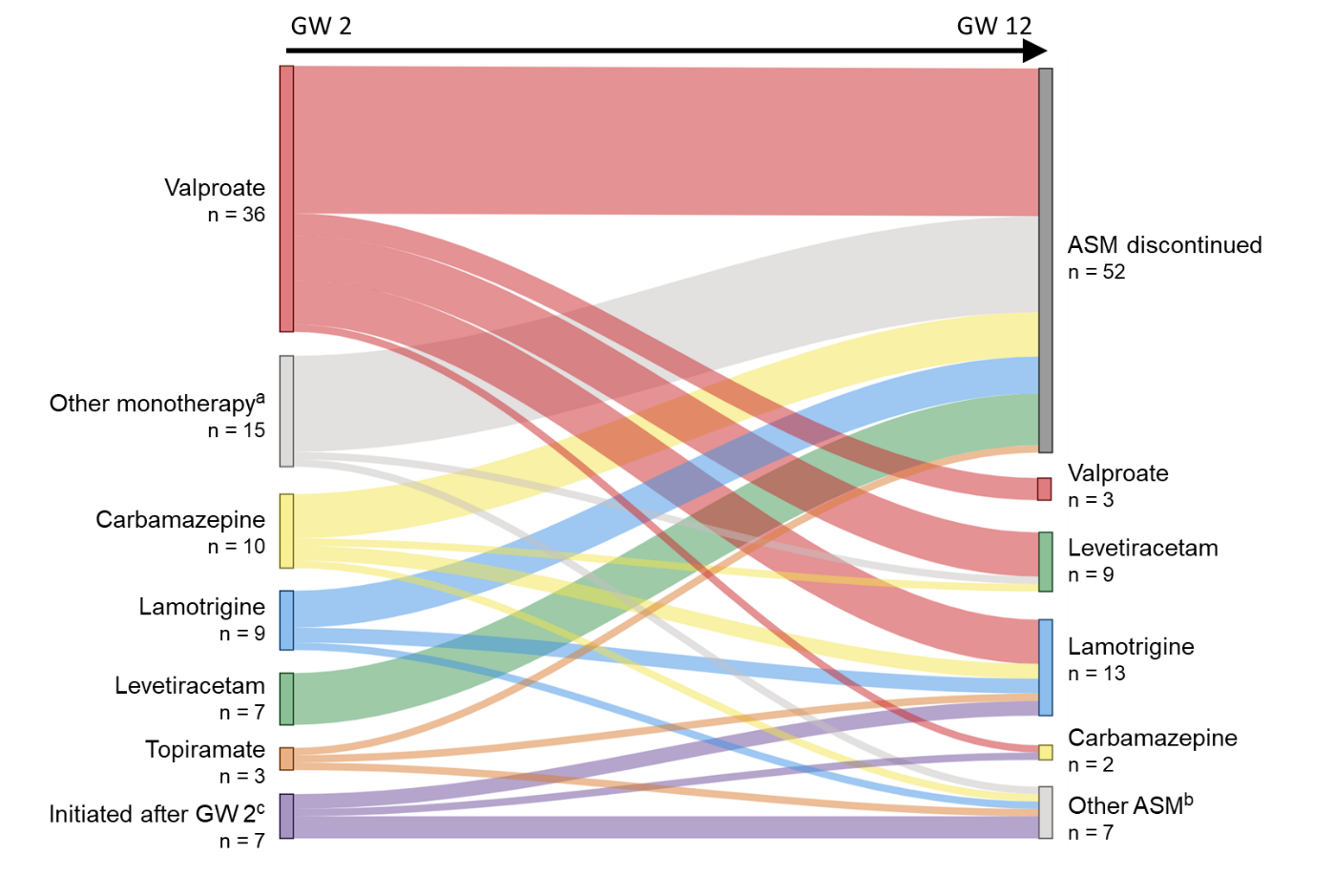


Figure S5: Treatment changes in women with initial monotherapy (n=80). Only women discontinuing or changing their initial treatment regimen until gestational week 12 are shown.

^a^ Other monotherapies (in GW 2): Oxcarbazepine (n=6), gabapentin, phenytoin or sultiame (each n=2) and phenobarbital, pregabalin or zonisamide (each n=1).

^b^ Other ASM (in GW 12): Clobazam, lacosamide, levetiracetam or oxcarbazepine (n=2).

^c^ Initiated after GW 2: Lamotrigine (n=2) and carbamazepine, clobazam, lacosamide, levetiracetam or oxcarbazepine (each n=1). An additional ASM until GW 12 was added in three women with valproate.

ASM, antiseizure medication; GW: gestational week

Graph created using [www.sankeymatic.com](http://www.sankeymatic.com)

Supplementary Table

## Table S1

The following ASMs were included for selection of exposed pregnancies

| Barbexaclone  Brivaracetam  Cannabidiol  Carbamazepine  Clobazam  Clonazepam  Eslicarbazepine  Ethosuximide  Gabapentin | Lacosamide  Lamotrigine  Levetiracetam  Mesuximide  Oxcarbazepine  Perampanel  Phenobarbital  Phenytoin  Pregabalin | Primidone  Retigabine  Rufinamide  Stiripentol  Sultiame  Topiramate  Valproate  Vigabatrin  Zonisamide |
| --- | --- | --- |
